# Evaluation of Epstein-Barr Virus Latent Membrane Protein 2 Specific T-Cell Receptors Driven by T-Cell Specific Promoters Using Lentiviral Vector

**DOI:** 10.1155/2011/716926

**Published:** 2011-09-28

**Authors:** Dongchang Yang, Qing Shao, Hua Sun, Xiaoxin Mu, Yun Gao, Runqiu Jiang, Jiajie Hou, Kun Yao, Yun Chen, Beicheng Sun

**Affiliations:** ^1^Liver Transplantation Center, The First Affiliated Hospital of Nanjing Medical University, Jiangsu Province, Nanjing 210029, China; ^2^Department of Ophthalmology, The First Affiliated Hospital of Nanjing Medical University, Jiangsu Province, Nanjing 210029, China; ^3^Brown Foundation Institute of Molecular Medicine, The University of Texas Health Science Center at Houston, Houston, TX 77030, USA; ^4^Department of Microbiology and Immunology, Nanjing Medical University, Nanjing 210029, China

## Abstract

Transduction of latent membrane protein 2 (LMP2)-specific T-cell receptors into activated T lymphocytes may provide a universal, MHC-restricted mean to treat EBV-associated tumors in adoptive immunotherapy. We compared TCR-specific promoters of distinct origin in lentiviral vectors, that is, V**β**6.7, delta, luria, and V**β**5.1 to evaluate TCR gene expression in human primary peripheral blood monocytes and T cell line HSB2. Vectors containing V**β** 6.7 promoter were found to be optimal for expression in PBMCs, and they maintained expression of the transduced TCRs for up to 7 weeks. These cells had the potential to recognize subdominant EBV latency antigens as measured by cytotoxicity and IFN-**γ** secretion. The nude mice also exhibited significant resistance to the HLA-A2 and LMP2-positive CNE tumor cell challenge after being infused with lentiviral transduced CTLs. In conclusion, LMP2-specific CTLs by lentiviral transduction have the potential use for treatment of EBV-related tumors.

## 1. Introduction

 Epstein-Barr virus (EBV) is a ubiquitous human herpesvirus associated with many human malignancies including a subset of Hodgkin disease, Burkitt lymphoma, nasopharyngeal carcinoma (NPC), and some gastric carcinomas [1–5]. The malignancies associated with EBV can be grouped into 3 types according to the latency gene expressional profiles [4–6]. In NPC, the EBV proteins expressed are EBNA1, latent membrane protein 1 (LMP1), and LMP2 [7]. It has been shown that all 3 antigens can induce CD8^+^ cytotoxic T lymphocytes (CTLs), which play roles in antitumor immune response [8, 9]. However, only weak responses against EBNA1 can be detected in some individuals and the phenotypic and functional analyses of these induced EBNA-1-specific T cells revealed that EBNA1 is presented to CD4^+^ T helper as well as Treg cells, which suppress the antiviral immune response. Moreover, the poor immunogenicity of EBNA1 has also been attributed to the presence of a Gly-Ala repeat (GAr) sequence, which prevents the presentation of EBNA1-derived antigenic peptides by MHC class I molecules. This GAr-mediated function has been linked to its capacity to prevent EBNA1 synthesis, as well as proteasomal degradation [10–13]. On the other hand, LMP-1 is the only EBV protein with recognized oncogenic activity that can transform normal cells into malignant ones, thereby limiting its application as a potential immunotherapeutic target. Additionally, the LMP1-specific CTL frequency is low, and the reactivation of LMP1-specific CTL lines has been shown very difficult, in part because LMP1 is toxic when expressed at high levels. In NPC, LMP2 offers the best opportunity for specific targeting since it is consistently expressed and the T-cell determinants in LMP2 sequence have been well defined [14–17]. Many studies, including clinical trials, have proven LMP2 to be an ideal immunotherapeutic target and inducer, which so far has not shown oncogenicity [16, 18–20]. It has been shown that this antigen can be processed by a proteasome system. The peptides are engaged in the major histocompatibility complex (MHC) class I, then move to the cell surface, and migrate to the CD8^+^ T cells on the cell surface [21–24]. Many CD8^+^ T cell-recognizing epitopes have been identified and most of them are conserved in NPC cells among different populations. Low LMP2 is a widely used targeting molecule and antigen for the immunotherapy of type II EBV-associated malignancies [19, 21, 25–29]. 

Adoptive immunotherapy with CTLs holds great promise for the treatment of cancer. Among them, the treatment of EBV-associated tumors has by far shown the most success [26–28, 30, 31]. With the development of molecular and cellular biology, tumor-specific CTLs can be selected and the T cell receptor genes can be cloned into highly efficient viral vectors for transfer into the patient's T cells. This concept has been utilized since 1999, when Clay et al. transferred lytic function by retroviral vectors encoding the *α* and *β* chains of the TCR against EBV-associated tumors [32]. Today many practitioners have designed and applied these engineered CTLs for the treatment of various human malignancies [33–37]. However, the efficacy and efficiency of this application still needs to be optimized, especially when using retro- or lentiviral vectors for TCR transduction. These vector systems can integrate transgenes into chromosomes that have the potential to “immortalize” a normal cell. Thus, a specific T-cell promoter that can be activated only in T cells becomes critical for safety concerns. At the same time, transducing efficiency should be considered when using both these viral vectors.

 In our investigation, we used an HLA-A2-restricted EBV LMP2-specific TCR, TCR5.05, to compare 4 different kinds of T-cell-specific promoters: Luria, Delta [38], V*β*5.1 [39], and V*β* 6.7 [40]. Our results show that all these T-cell-specific promoters can drive the transcription of the TCR gene without changing the transduced T cell phenotypes. We also found that CTLs generated by a lentiviral vector containing specific promoters and TCR genes can lyse target cells specifically. We further evaluated the CTLs in vivo and found that they can retard the growth of EBV-LMP2 expressing tumors and prolong the life of tumor-bearing mice. We reported for the first time that V*β* 6.7 promoter is most efficient when using the lentiviral vector to transduce T cells for targeting HLA-A2-restricted EBV-LMP2 antigens. This study may be helpful in designing and developing novel TCR-based adoptive immunotherapy for the treatment of EBV-associated tumors.

## 2. Material and Methods

### 2.1. Animals and Cell Lines

 Six- to 8-week-old nude mice were purchased and maintained in the SPF animal facility at Nanjing Medical University. All procedures used in this study complied with institutional policies of the Animal Care and Use Committee of Nanjing Medical University.

The cell lines used in these experiments included 293T, HSB2 (human leukemic T-cell line); HLA-A2 restricted, Epstein Barr virus-transformed B lymphoblastoid cell lines (LCLs); K562; CNE (nasopharyngeal carcinoma cell line); T2 cell lines (deficient in TAP but still express low amounts of MHC class I on the surface of the cells, kept in the author's lab). In animal model, CNE cells stably expressing HLA-A2 and LMP2 were established by plasmid pIRES/HLA-A2/LMP2 transfection and selection. All cell lines were cultured in RPMI 1640 plus 10% fetal bovine serum, L-glutamine, nonessential amino acids, and penicillin-streptomycin (100 U/mL) (Invitrogen).

### 2.2. Construction of Lentiviral Vector Plasmids

 TCR plasmid PL5.05 and 4 T lymphocyte-specific promoters (PSK-Delta/V*β* 5.1/Luria/V*β* 6.7) were kindly provided by Rimas Orentas from Medical College of Wisconsin. EBV LMP2-specific TCR cDNA we used was obtained from T-cell clone which was generated by incubating HLA-A2-restricted PBMC with peptide (CLGGLLTMV, LMP2: 426–434) as described by Orentas et al. [36]. TCR PL5.05 *α* and *β* chains were amplified by PCR using PL5.05 as a template and cloned into 4 lentiviral vectors containing various 4 T-cell promoters constructed from the parent PWPT-GFP vector. The primers were *α* chain: Primer1A: CAACGCGTCGGAATTCAGGCTCTCTTG; Primer2A-3A: GTCATCGTCTTTGTAGTCGCTGGACCACAGCCGC;   CAGGTCGACTCACTTGTCGTCATCGTCTTTGTAGT;*β* chain: Primer1B: ACTACGCGTCACCATGGCTATAGTGTCTCTAGATCAAAG; Primer2B-3B: TTCTGAGATGAGTTTTTGTTCCTAAAGGGAACAAAAGCTGGAAGTCGACTCAATTCAGATCCTCTTCTGAGATGAGTTT. The *α* and *β* were linked with Flag and Myc tag, respectively. All the amplicons were sequenced and cloned into Mlu I and Sal I sites of PWPT-GFP vectors.

### 2.3. Lentivirus Production

 Lentiviruses were prepared by transient transfection of 293T cells, using a liposomal cotransfection method. To summarize, the 293 T cells were seeded at 1 × 10^7^ cells per 10-cm plate. The cells were transfected 12–16 hours later with 20 *μ*g lentiviral transfer vector, 12 *μ*g Delta 8.9, and 18 *μ*g VSV-G envelope plasmids 8.91 (Delta 8.9 and VSV-G envelope plasmids are helper lentiviral plasmid which are used for packing lentivirus). Forty-eight to 72 hours later, the supernatant was collected, centrifuged to remove the cellular debris, and concentrated approximately 30-fold by ultracentrifugation.

### 2.4. Determination of Lentiviral Titer

 Titers of concentrated lentivirus encoding green fluorescent protein (GFP) were determined by serially diluting and infecting 293T cells by the polybrene transduction method as previously described [41]. Titers (transducing units (TUs) GFP-positive cell dilution factor) of the lentiviral vectors ranged from 10^6^ to 10^7^ TU/mL.

### 2.5. Transduction of PBMCs and T Cells

 Peripheral blood monocytes (PBMCs) were from an HLA-A2, healthy human. T cells were obtained from anti-CD3 conjugated magnetic beads (Miltenyi Biotec, Bergish Glad-bach, Germany). The PBMCs and T cells were cultured in AIM-V and interleukin-2 (IL-2; PeproTech, Rocky Hill, NJ, USA) at 300 IU/mL. For OKT3 stimulation, the cells were placed initially in either a medium with anti-CD3 antibody, OKT3 (Ortho Biotech, Bridgewater, NJ, USA) at 50 ng/mL or in an OKT3 medium after transduction at the initial changing of the culture medium in the presence of IL-2. For transduction of the PBMCs or T cells, 1 × 10^6^ cells were adjusted to a final volume of 1 mL in a 24-well, tissue culture-treated plate with the viral supernatant and Polybrene (8 mg/mL; Sigma, St. Louis, Mo, USA). The cells were transduced by centrifugation of the plates at 1000 g for 1.5 hours at 32°C. The plates were placed in a 37°C, humidified, 5% CO_2_ incubator overnight, and the medium was replaced the next day.

### 2.6. Flow Cytometric Analysis

 CD3 expression on cell surface was measured with allophycocyanin-conjugated antibodies and the corresponding isotype controls (BD Biosciences). TCR PL5.05 staining was performed by using anti-TCR *α* chain antibody (prepared from our lab) followed by phycoerythrin (PE)-labeled second antibody. Cells were stained in a FACS buffer made of PBS (Invitrogen, Carlsbad, Calif, USA) and 0.5% bovine serum albumin. Cells were collected with a FACSCalibur flow cytometer (BD Immunocytometry Systems, San Jose, Calif, USA) and analyzed using CellQuest software (BD Biosciences).

### 2.7. Real-Time PCR

 After 3 days, total RNA was extracted from the HSB2 cells which have been infected with lentivirus containing EBV-LMP2-specific TCR *α* and *β* chain driven by Luria, Delta, V*β* 5.1, and V*β* 6.7 T-cell-specific promoters. cDNA was reverse transcripted by a high-capacity cDNA reverse transcription kit (ABI, Foster, Calif, USA) using random primers. For *α* chain SYBR forward primer: 5′-ctttcaaaacctgtcagtgattgg, reverse primer: 5′-cagcgtcatgagcagattaaacc. For *β* chain SYBR forward primer: 5′-ggccaccttctggcagaac, reverse primer: 5′-agagcccgtagaactggacttg. Real-time PCR with SYBR dyes was performed on an ABI 7900 real-time machine and analyzed by SDS2.4 software.

### 2.8. Western Blotting

 Fifty micrograms of total protein from each sample was loaded for SDS-PAGE and subsequently transferred onto the PVDF membranes. After blocking, the membranes were hybridized with anti-Flag and Myc tag antibodies, respectively. The membranes were washed and incubated with secondary antibody, followed by developing.

### 2.9. Measurement of Lymphocyte Antigen Reactivity

Target cells were prepared by using T2 cells pulsed with peptides (10 ng/mL) in cell culture medium or tumor cell lines for 2 hours at 37°C and then washed twice in PBS. CD8^+^ T cells were isolated from lentiviral transduced PBMCs using anti-CD8 beads from Miltenyi Biotec according to the manufacturer's protocol. For the assay, effector cells (CD8^+^ T cells) and target (peptide-pulsed T2 or tumor cells) were incubated in a 0.2-mL culture volume in the wells of a 96-well culture plate at E : T = 50 : 1, 25 : 1, and 5 : 1. The cells were cocultured for 18 hours, and the supernatant was harvested. The supernatants were analyzed for interferon (IFN)-*γ* secretion, using a commercially available enzyme-linked immunosorbent assay (ELISA) kit (Bender Medsystem, Vienna, Austria). The supernatants were also measured for LDH levels using a commercially available kit (Roche, Boehringer Mannheim, Germany).

### 2.10. Mouse Immunization and Tumor Challenge

 Tumor-bearing model were established by injecting 1 ×10^6^ HLA-A2 and LMP2-positive CNE cells subcutaneously in the flank of six- to 8-week-old nude mice. The mice were infused with transduced CTL via tail vein 1 week after tumor cell injection weekly for a total of two weeks. Mice immunized with the mock or saline were used as a control. Tumor diameter was measured by calipers twice per week and recorded as the mean of narrowest and longest surface length for each animal in the group. Mice were sacrificed when the tumor size reached a 20 mm average diameter. Each experiment was performed at least twice, and results were essentially similar unless described otherwise.

### 2.11. Statistical Analysis

Data are expressed as mean ± standard error of mean (SEM), as indicated in each experiment. And the comparisons between the groups were made by one-way ANOVA followed by unpaired *t*-test. A 4.0 version (2005) of the GraphPad Prism software was used for this purpose. Values of *P* < 0.05 were considered significant. Survival curves were estimated using the Kaplan-Meier method, and significance was assessed using the log-rank or the *χ* ^2^ test.

## 3. Results

### 3.1. V*β* 6.7 Promoter Is the Most Optimal for TCR Expression

**Figure 1:**
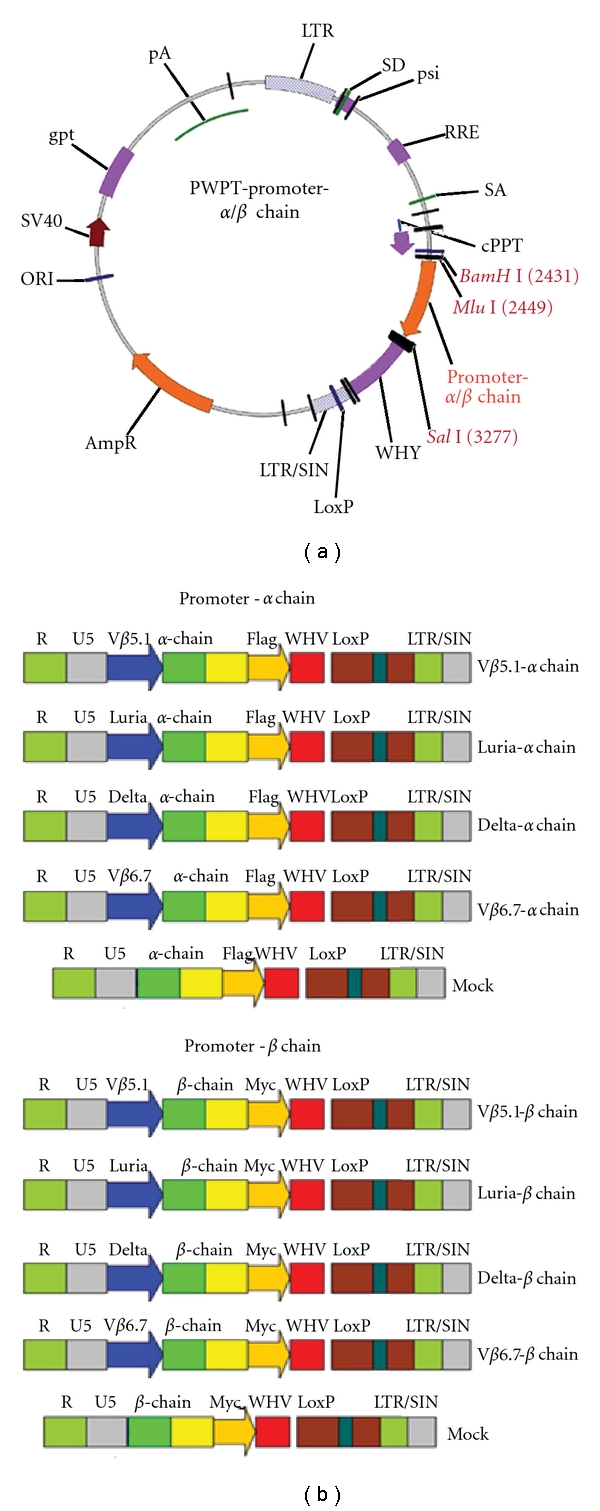
The map of lentiviral vector pWPT-promotor-*α*/*β* chain and the schematic diagrams representing the structures of the lentiviral vectors. (a) The map of lentiviral vector pWPT-promotor-*α*/*β* chain. The promoter-*α*/*β* chain was amplified by PCR and inserted between *Mlu *I and *Sal *I sites. (b) The schematic diagrams representing the structures of the lentiviral vectors. All the *α*/*β* chains of the anti-LMP2 TCR PL5.05 were driven by individual T-cell-specific promoter except mock which contains only *α* or *β* chains without any promoter region. Promoter-*α* chains in diagram forms were the lentiviral vectors designed to express *α* chain driven by V*β* 5.1, Luria, Delta, and V*β* 6.7 promoters, respectively. Promoter-*β* chain used V*β* 5.1, Luria, Delta, and V*β* 6.7 promoters to produce the individual *β* chain.

**Figure 2:**
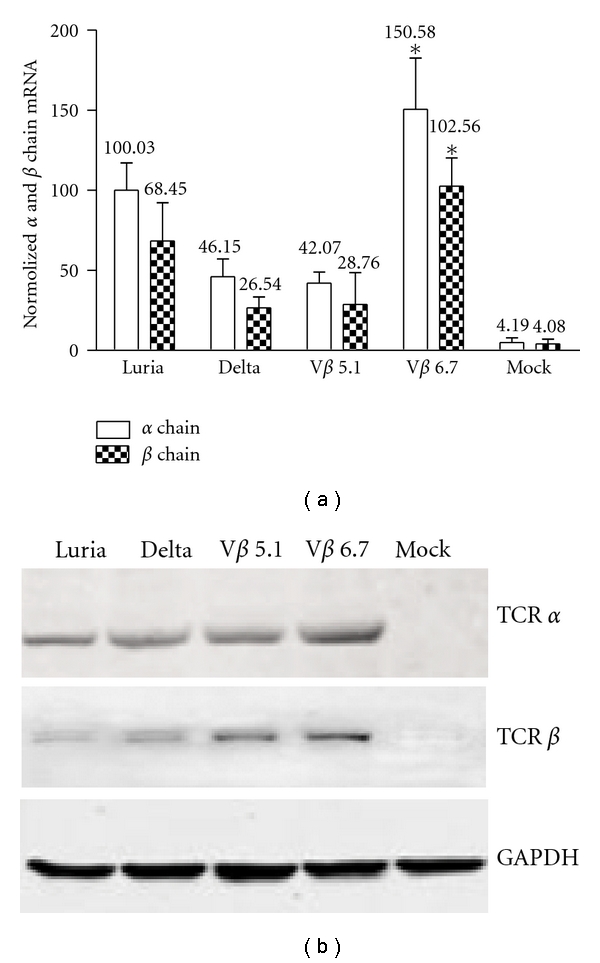
Comparison of lentiviral vector constructions driven by different T-cell-specific promoters. (a) Comparison of mRNA level of TCR PL5.05 *α* and *β* chain under different T-cell-specific promoters by using quantitative RT-PCR. The number on each column corresponds to the mean number of mRNA normalized by GADPH mRNA, and the vertical bar represents the SD. (b) Conventional western blot assays from HSB2 cells which are infected with lentivirus containing TCR *α* and *β* chains driven by V*β* 6.7, Delta, V*β* 5.1, and Luria promoters. Either Flag or Myc fusion proteins were transferred to membranes and incubated with the indicated antibody. Approximately, fifty micrograms of each protein per lane were applied for electrophoresis. Equal protein loading was controlled by staining of GAPDH (lower panel). Statistical analysis was determined using the Student *t*-test with *P < 0.05, compared to other groups.

 The map of lentiviral vector pWPT-promotor-*α*/*β* chain and the schematic diagrams representing the structures of the lentiviral vectors are shown in Figure 1. The promoter-*α*/*β* chain was amplified by PCR and inserted between *Mlu*I and *Sal*I sites. The mock vector contains TCR *α*/*β* chain without any T-cell-specific promoter. TCR expression under four T-cell-specific promoters was compared by using real-time PCR, as shown in Figure 2(a). The HSB2 cells were incubated for 24 hours in medium and then exposed to each vector at a multiplicity of infection (MOI) of 10. Three days aftertransduction, the T cells were analyzed by real-time PCR and Western blotting. We observed that all the lentiviral vectors were able to transduce the T cells, using the Luria, Delta, V*β* 5.1, and V*β* 6.7 promoter-containing vectors. The V*β* 6.7 promoter vector had the highest TCR at transcriptional level. When the normalized *α* and *β* chain mRNA levels of the Luria promoter group were set at 100.03 ± 21.09 and 68.45 ± 23.75, Delta was 46.15 ± 11.01 and 26.54 ± 6.86, V*β* 5.1 was 42.08 ± 6.78 and 28.76 ± 19.75, and V*β* 6.7 was 150.58 ± 32.02 and 102.564 ± 17.75, and mock was 4.89 ± 3.09 and 4.08 ± 2.98. The mRNA levels of TCR were consistent with the protein levels used in Western blotting to detect the protein levels of the TCR *α* and *β* chain. Protein levels of TCR were much higher in the V*β* 6.7 group than in the other groups (Figure 2(b)). The expression of TCR *α* and *β* chain on the HEK293T cells and HepG2 cells (human hepatocellular carcinoma cell line) were almost not detected (Data not shown). These results suggest that the lentiviral vectors can express TCR in the T cell lines and PBMCs. Four different promoters have different levels of capacity to drive TCR expression. 

### 3.2. Lentiviral Vectors with Various Promoters Can Transduce T Cells Efficiently

**Figure 3:**
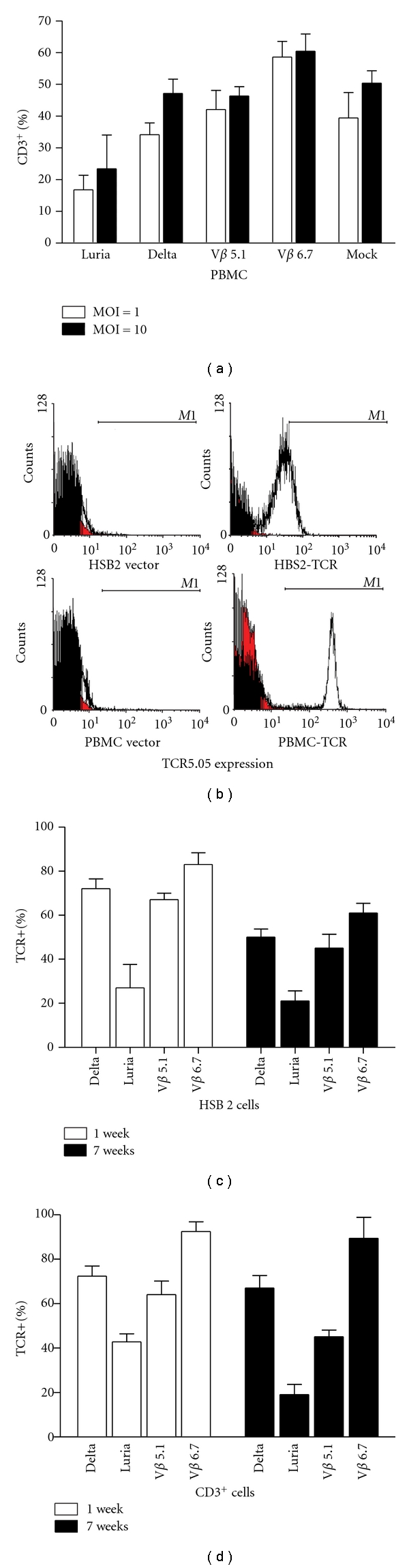
Expression of TCR *α* and *β* chains in the HSB2 cells and PBMCs. (a) The PBMCs were incubated with lentivirus containing EBV-LMP2 TCR *α* and *β* chains driven by T cell-specific promoters at MOIs of 1 and 10. Three days aftertransduction, cells were collected and stained for CD3 mobilization as a measure of TCR *α* chain expression. (b) Photographs of flow cytometric analysis for TCR *α* chain expression in HSB2 cells (top) and PBMC cells (bottom). The cells were infected with recombinant lentivirus or control empty lentivirus at MOI of 10 for 3 days. TCR staining was performed by using anti-TCR *α* chain antibody (prepared from our lab) followed by PE-labeled second antibody. (c) Percentage of TCR *α* chain-positive cells in transduced HSB2 cells 1 week and 7 weeks after infection at MOI of 10. (d) Percentage of TCR *α* chain-positive cells in transduced CD3^+^ cells 1 week and 7 weeks after infection. PBMCs stimulated with IL-2 plus OKT3 for 24 hr were infected with the lentivirus at an MOI of 10. After 1 week, the PBMCs were analyzed by FACS for TCR *α* chain expression and then maintained in culture with IL-2 for 7 weeks for reanalysis for TCR *α* chain-positive cells.

 HSB2 and PBMCs were infected with lentiviral vectors having various promoters expressing the TCR *α* and *β* chain at MOI = 1 or 10. Three days after infection, expression of the TCR *α* chain was detected in the CD3^+^ T cells by FACS with a Flag tag antibody. At MOI = 1, the TCR *α* chain positivities from CD3^+^ cells were 16.76 ± 4.62%, 34.15 ± 3.71%, 42.08 ± 6.03%, and 58.58 ± 5.02% under Luria, Delta, V*β* 5.1, and V*β* 6.7, respectively. At MOI = 10, the positive TCR *α* chain was 23.42 ± 10.63%, 47.14 ± 4.53%, 46.33 ± 2.96%, and 60.46 ± 5.41%, under Luria, Delta, V*β* 5.1, and V*β* 6.7 T-cell-specific promoters of CD3^+^ cells, respectively (Figure 3(a)). The V*β* 6.7 group had the highest transducing efficiency, as evidenced by means of 58.58% and 60.46% positive at MOI = 1 or 10. We next checked the LMP2-TCR expression by flow cytometric analysis. As shown in Figure 3(b), 51.3% or 62.1% of the HSB2 or PBMC cells, respectively, were positive when confirmed by the result of FACS. The empty lentiviral vector-infected control group showed no TCR 5.05 expression. To test the stability of TCR expression on the surface of the cytoplasm membranes, we checked the expression of TCR 1 and 7 weeks after transduction by using FACS. Our experiments showed that there were no significant changes in the TCR expression levels in either the HSB2 cells or the CD3^+^ T cells transduced by all 4 lentiviral vectors. However, the Luria group had the lowest, and the V*β* 6.7 group had the highest transduction efficiency 1 week and 7 weeks after transduction (Figures 3(c) and 3(d)). The percentage of TCR-positive cells in CD3^+^ group 1 week after transduction is similar to that of the 3-day transduction experiment described above. 

### 3.3. Transduced PBMCs Can Specifically Lyse HLA-A2/LMP2, Expressing Target Cells

**Figure 4:**
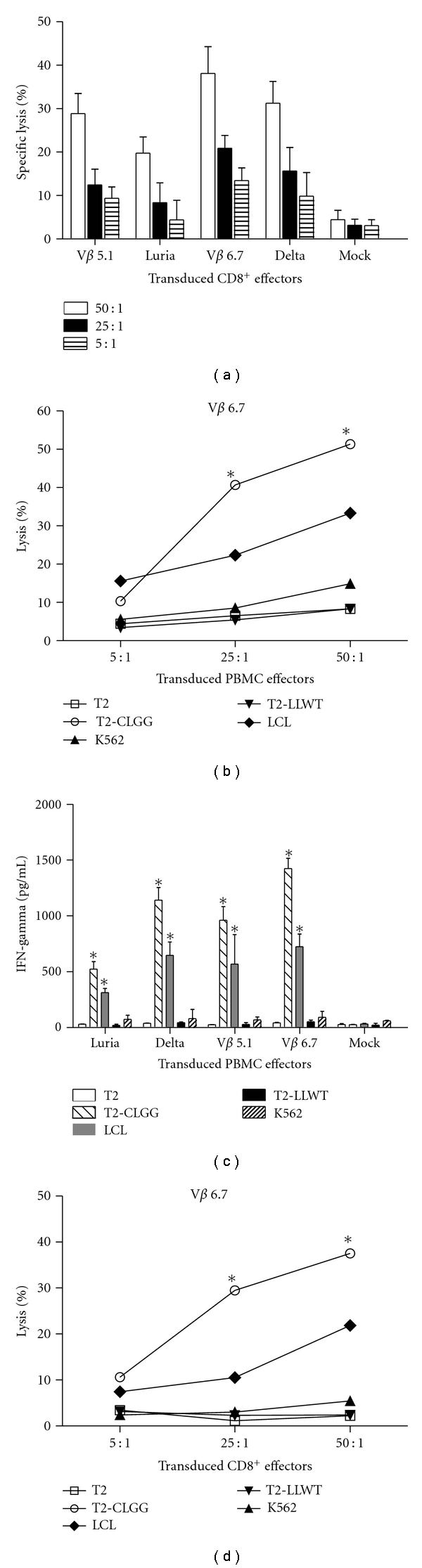
Transduced PBMCs specifically lyse HLA-A2-restricted LMP2-expressing target cells. (a) Lytic activity of CD8^+^ cells selected from PBMCs transduced with lentivirus containing LMP2-TCR at indicated effector-to-target cell ratios (E : T) was demonstrated in an LDH-release assay. Targets were HLA-A2-restricted T2 cells loaded with CLGG. Results are expressed as percent of the value measured in control cells incubated with the same volume of medium (mean ± SD of 3 replicates). Lytic activity of PBMCs (b) or CD8^+^ cells (d) selected from PBMCs transduced with lentivirus-containing LMP2-TCR driven by T-cell promoter V*β* 6.7 at indicated E : T was demonstrated in an LDH-release assay. Targets were HLA-A2-matched T2, T2-CLGG, LCL, T2-LLWT, and exceptional K562 cells. All figures are representative of 3 or more experiments using the same PBMC donor. (c) Levels of IFN-*γ* being released into the media from transduced PBMC effectors in the lysis assay above. PBMCs expressing LMP2-TCR were cocultured for 16 hr with the indicated target cells. The concentration of IFN-*γ* secreted into the medium was measured in an ELISA kit.

 To assess the recognition of tumor antigens by lentivirus-transduced PBMCs and CD8^+^ T cells, the cells were cocultured with the indicated tumor cell lines or T2 cells pulsed with LMP2_426–434_ (CLGGLLTMV) (CLGG). After sorting, the CD8^+^ cells were collected and incubated with target cells at effector-to-target-cell ratios (E : T) = 50 : 1, 25 : 1, and 5 : 1. As shown in Figure 4(a), the V*β* 6.7 group has the highest lytic activity when using all 3 E : T ratios. To test the specificity of cytotoxicity, we chose the V*β* 6.7 lentiviral vector infected with PBMCs and CD8^+^ groups against different targeting cells. The results showed that V*β* 6.7 lentiviral vector-infected PBMCs could lyse T2-CLGG and LCLs effectively moderately but could not lyse T2 cells, T2 cells loaded with nonrelated peptides (T2-LLWT), and K562 cells (Figure 4(b)). 

We also measured the IFN-*γ* levels in the supernants of the transduced-PBMC cytotoxicity experiments. All 4 promoter-containing lentiviral vector groups which transduced PBMCs secreted high levels of IFN-*γ* (>500 pg/mL) when incubated with CLGG and LCLs but secreted very low levels of IFN-*γ* when incubated with T2, T2 LLWT, or K562 cells (Figure 4(c)). These results further confirmed that the lysis is specific. 

We next tested the cytotoxicity of V*β* 6.7-transduced CD8^+^ T cells against the targeting cells described above. Similar to the result involving PBMCs, the transduced CD8^+^ T cells had a higher cytotoxicity against the T2-CLGG and LCL, but minimal effects on T2-LLWT, T2, and K562 cells (Figure 4(d)). LCLs are EBV-transduced B lymphocytes which belongs to type III infection, expressing nine EBV genes encoded by the virus including LMP2. The results indicated that V*β* 6.7 lentiviral transduced T cells can specifically lyse HLA-A2-restricted tumor cells expressing EBV-LMP2. 

## 4. Transduced CD8^+^ Cells Can Slow the Growth Rate of LMP2-Expressing CNE Tumors in Mice

**Figure 5:**
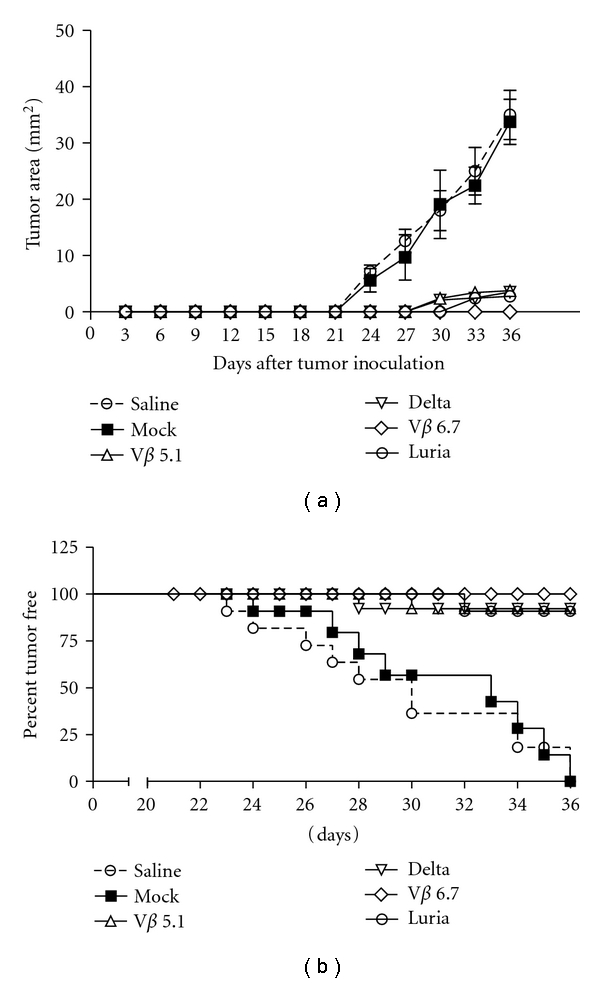
Significantly reduced tumor burden and enhanced tumor-free survival in nude mice implanted with LMP2-expressing tumors after infused with transduced T cells. (a) Tumor mouse model was established as described. After 7 days, mice were infused with different lentiviral transduced T cells twice at a 1-week interval. PBS-immunized mice were used as control. Tumor growth was recorded twice a week. Tumor sizes are expressed as the average of two perpendicular diameters of the tumor. Graphs show mean ± SE; *P < 0.05; *n* = 8/group. (b) Comparison of survival times of tumor-bearing mice infused with T cells ex vivo transduced by lentivirus containing LMP2-TCR *α* and *β* chains driven by various T-cell promoters. Tumor burden was monitored on a weekly basis. Significant differences were observed for all transduced T-cell vaccination group. *P < 0.01; *n* = 14/group. Note: tumor growth rates and survival times were discontinued when the tumor reached 1 cm^2^ and the mouse was sacrificed.

CNE tumor cells stably expressing HLA-A2 and LMP2 were inoculated subcutaneously at 5 × 10^5^ cells per mouse to establish the tumor model. Ten days later, the peptide-pulsed, lentiviral vector-transduced CD8^+^ cells were infused via the tail vein. The tumors were monitored daily till the tumor reached 1 cm^2^, when the mouse was sacrificed. Each group of the transduced CD8^+^ cells was shown to slow or abolish the established tumors in the mouse model (Figure 5(a)). There were no statistically significant differences between the antitumor effects of the 4 promoter groups. All immunized groups were significantly different when compared with the saline and mock groups (Figure 5(b)). The mice were deemed dead when the tumor reached 1 cm^2^. None of the mice in the V*β* 6.7 group died, and only 1 mouse died in each of the Luria, Delta, and V*β* 5.1 groups. All the mice in the saline group died 36 days after inoculation. These results demonstrated the therapeutic effects of reinfused CTL transduced with lentiviral vectors containing the specific TCR.

## 5. Discussion

Adoptive T-cell immunotherapy remains an active area in the correction of birth defects and the treatment of malignancies [26–28, 30, 31]. Unlike traditional immunotherapeutic approaches such as use of vaccine or antitoxin, adoptive T-cell immunotherapy is specific, repeatable, and much more effective. Adoptive T-cell therapy has advanced from simple ex vivo expansion of therapeutic T cells to gene-modified T cells. As the most important functional molecule of T cells, specific TCR has been cloned from effective and specific T-cell clones and transduced into modified T cells, which may express a large quantity of cytokines or costimulating receptors to boost function of the T cells [42–47]. 

The EBV-associated tumor is a potential target for adoptive T-cell immunotherapy because of its latent antigen expression profile. Orentas et al. reported that, by using SAMEN retroviral vector, they could demonstrate the ability to transfer CTL activity from an LMP2 peptide-specific CTL clone to a stimulated PBMC population. These TCR-transduced PBMCs showed specific immunoactivity against LMP2 targets [36]. Here, we continued this work and attempted to develop an effective lentiviral-based TCR transduction system for future clinical practice. Compared with retroviral vectors, lentiviral vectors have many advantages including the ability to transduce minimally stimulated PBMCs, and they have a potentially safer integration site preference [48, 49]. Our results showed that lentiviral vectors can effectively transduce PBMCs and CD3^+^ cells with LMP2-specific TCRs using 4 different T-cell-specific promoters. 

Using highly active T cell promoters to drive TCR *α* and *β* chains has been reported by many groups to evaluate different combinations of promoters. It has been shown to express that multiple protein subunits, viral vector, and promoters are required intensive optimization [50, 51]. We used LMP2-specific TCRs to compare 4 different promoters in lentiviral vectors. TCR *α* and *β* chains are driven by each promoter independently. Our results showed that, although lentiviral vectors of the various promoters express TCR *α* and *β* chains at different levels, all groups of transduced CD8^+^ cells dramatically slowed or abolished the growth of LMP2-positive tumors. These results indicate that the transducing efficiency of lentiviral vectors containing different promoters does not affect the antitumor activity of CTLs. In future studies, we hope to emphasize the expansion of functional CTLs after selection rather than switching promoters to achieve higher transduction efficiency. 

We have demonstrated that, for a single promoter, V*β* 6.7 is relatively superior to other promoters. Since our work solely compared T-cell-specific promoters, we could not exclude the possibility that others may have more powerful functions. Jones et al. generally compared specific and nonspecific promoters, which gave a comprehensive picture of promoter selection and combination [51]. We believe that the trend of adoptive T-cell immunotherapy is to develop safer and more effective vectors to engineer T cells. The priority is still safety. A specific T-cell promoter can limit the expression of transgenes in a relatively small subset of cells, so it is theoretically safe. Our study provides suggestions for future designing of lentiviral vectors in adoptive T-cell immunotherapy. 

##  Conflict of Interests

The authors have contributed significantly and declare that they have no conflict of interests.
